# A Whole-Food-Based Health Product (A-F Betafood^®^) Improves Gallbladder Function in Humans at Risk of Gallbladder Insufficiency: A Randomized, Placebo-Controlled Clinical Trial

**DOI:** 10.3390/nu12020540

**Published:** 2020-02-20

**Authors:** Malkanthi Evans, Najla Guthrie, Bassem F. El-Khodor, Brandon Metzger, Saradhadevi Varadharaj

**Affiliations:** 1 KGK Science, London, ON N6A 5R8, Canada; 2 Standard Process Inc, Palmyra, WI 53156, USA

**Keywords:** A-F Betafood^®^, beets, randomized, double-blind, placebo-controlled, gallbladder condition, gallbladder function, liver function

## Abstract

A-F Betafood^®^ is a whole food-based health product. The product contains phytonutrients and bioactives with antioxidant properties that may support gallbladder and liver function. Herein, we investigated the efficacy of A-F Betafood^®^ on gallbladder and liver function. In this randomized, placebo-controlled, parallel study fifty overweight but otherwise healthy adults received A-F Betafood^®^ or placebo for 12 weeks. Gallbladder function as assessed by gallbladder volume, ejection fraction (GBEF), ejection rate, wall thickness and liver function determined via aspartate aminotransferase, alanine aminotransferase, and gamma-glutamyltransferase, and high-sensitivity c-reactive protein analysis at baseline and week 12 were the primary outcomes. Total cholesterol, low-density lipoprotein-cholesterol, high-density lipoprotein-cholesterol, triglycerides, and oxidative stress markers including oxidized low-density lipoprotein, tumor necrosis factor-α, adiponectin and malonyldialdehyde (MDA) were assessed as secondary outcomes. A-F Betafood^®^-supplementation significantly reduced gallbladder wall thickness (*p* = 0.049) by 9% compared to placebo from baseline to week 12. The A-F Betafood^®^ group alone had significant improvements in gallbladder volume (32%; *p* = 0.044) and GBEF (19%; *p* = 0.047) at week 12. There were no changes in liver function, oxidative stress markers or blood lipid concentrations, though MDA concentrations decreased in both groups. Our findings demonstrate A-F Betafood^®^-supplementation significantly improves measures of gallbladder function and support healthy gallbladder function in the individuals with gall bladder condition.

## 1. Introduction

Gallbladder disease is a significant health burden in the developed world and its prevalence has risen by more than 20% in the past three decades [1]. In the United States, 20–25 million individuals are affected by gallbladder disease, resulting in an average annual treatment cost of $6.2 billion [2]. Obesity, diet, rapid weight loss, metabolic syndrome, and conditions such as cirrhosis, Crohn’s disease, irritable bowel syndrome, and gallbladder stasis increase the risk of gallstone formation [3]. Surgical removal of the gallbladder, cholecystectomy, is the most recommended treatment for gallbladder disease; however, the procedure increases the risk of non-alcoholic fatty liver disease, cirrhosis and metabolic effects [4]. Oral dissolution therapy using ursodeoxycholic acid is currently one of the leading non-invasive alternative treatments for gallstones. Ursodeoxycholic acid dissolves small cholesterol gallstones by decreasing hepatic secretion of cholesterol and lowering the chances of unsaturated gallbladder bile formation [5]. Although oral dissolution therapy has proven to be a safe alternative to cholecystectomy, it has a gallstone recurrence rate of 50%–70% at 12 years post-treatment [6]. 

The high incidence rate and treatment cost associated with gallbladder disease have encouraged the search for preventative strategies. In approximately 75% of cases, impaired gallbladder motility results from the accumulation of cholesterol gallstones. Therefore, agents that reduce lipid concentrations or improve gallbladder motility may reduce the need for surgical interventions and/or the use of potentially harmful pharmaceutical products [7]. 

In humans, dietary components have been shown to significantly improve gallbladder function and motility. Consumption of coffee increases gallbladder contractility and cholecystokinin release to facilitate digestion in the small intestine [8] and decreases cholesterol crystallization in bile [9]. Vitamin C supplementation has been shown to reduce the risk of gallstone disease by lowering bile acid formation [10]. Betalains and betaine, naturally occurring phytonutrients, have also been suggested to promote liver and gallbladder function [11]. Betanin, a well characterized betalain found in beetroot, shows 1.5- to 2.5-fold higher oxidant-scavenging activity compared to similar molecules [12]. In addition, betanin bound low-density lipoprotein (LDL) was found to increase LDL resistance to copper-induced oxidation [13]. Betaine, on the other hand, acts as both an osmolyte to protect cells under stress and a lipotrope to prevent and reduce fat accumulation in the liver [14]. These findings highlight the potential of these agents to promote gallbladder function and prevent gallbladder insufficiency. 

The current study evaluated the efficacy of A-F Betafood^®^, a whole food-based health product, on gallbladder function using ultrasound measurements of gallbladder wall thickness, volume, gallbladder ejection fraction (GBEF), and gallbladder ejection rate (GBER). Furthermore, liver function was assessed using aspartate aminotransferase (AST), alanine aminotransferase (ALT), and gamma-glutamyltransferase (GGT) as markers. High sensitivity c-reactive protein (hs-CRP) was measured to evaluate inflammation, along with inflammatory cytokine tumor necrosis factor (TNF)-α, and oxidative stress markers oxidized low-density lipoprotein (oxLDL), adiponectin and malonyldialdehyde (MDA). The current study also investigated changes in circulating lipid concentrations, in the form of total cholesterol, low-density lipoprotein cholesterol (LDL-C), high-density lipoprotein cholesterol (HDL-C), and triglycerides. We hypothesized that a 12-week supplementation protocol with A-F Betafood^®^ would improve gallbladder and liver function. 

## 2. Materials and Methods

### 2.1. Study Design

**Figure 1:**
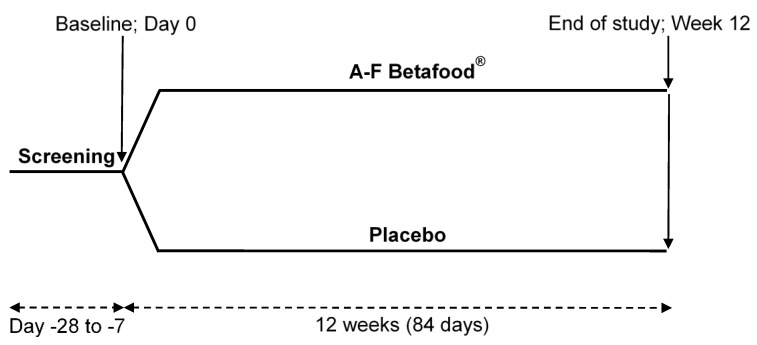
Schematic representation of clinical trial design. Fifty participants were enrolled in a 12-week double-blind, randomized, placebo-controlled, two-arm parallel study. All participants enrolled in the study completed all study related assessments at baseline (Day 0) and 12 weeks post-supplementation.

A 12-week randomized, double-blind, placebo-controlled parallel study was conducted at multiple centers, KGK Science Inc., London, ON, Canada, KGK, Orlando, FL, USA and Westside Family Medical Center, Kalamazoo, MI, USA from December 2013 to February 2018 (Figure 1). The study was performed according to the Declaration of Helsinki guidelines, and was approved by the Therapeutic Products Directorate (TPD), Health Canada, Ottawa, ON, Canada; the Institutional Review Board (IRB), Quorum Review, Seattle, WA, USA. All participants provided written informed consents and were compensated for time spent (Clinicaltrials.gov: NCT01981343). 

### 2.2. Subject eligibility

The inclusion criteria were: healthy males and females between the ages of 40–75 years, BMI of 25–32.5 kg/m^2^, resting heart rate of 50–80 bpm, family history of gallbladder condition or previous history of gallbladder attacks, and gastrointestinal distress with fatty foods. Exclusion criteria were: pregnant and breastfeeding women, presentation with cholecystitis, gallstones, or gallbladder wall thickness >3 mm as determined by ultrasound, use of prescription medications or natural health products that affect gastric/gastrointestinal function within 4 weeks prior to randomization, uncontrolled hypertension defined as untreated systolic blood pressure >160 mm Hg and/or diastolic blood pressure >100 mm Hg, allergy or sensitivity to test product ingredients, cognitive impairments or unstable psychiatric disorders requiring hospitalization within 6 months prior to enrolment, and any other condition that may adversely affect the participant’s ability to complete the study or may pose a significant risk to the participant as determined by the Medical Director. 

At screening (up to 4 weeks prior to baseline) participants had vital sign and anthropometric measurements taken and provided blood samples to assess clinical chemistry and hematology values. If all measures were within normal clinical ranges, the participant was deemed healthy and eligible to complete the study.

### 2.3. Intervention

Participants consumed either (i) A-F Betafood^®^ tablets which contained beet leaf (26.23% w/w), carrot powder (25.09%), beet powder (15.58%), calcium lactate (7.71%), wheat germ (7.38%), magnesium citrate (3.61%), bovine liver (2.29%), bovine prostate (2.29%), yeast (2.29%), vitamin A palmitate (2.13%), bovine kidney (2.13%), alfalfa (1.31%), flaxseed (0.56%), orchic extract (0.62%), ascorbic acid (0.20%), iodozein (0.16%), pyridoxine (0.16%), liver (0.14%), lecithin (0.06%), and vitamin E (0.06%), or (ii) an appearance- and taste-matched placebo (tapioca, sesame flour, pumpkin protein and honey) tablet. Participants consumed 2 tablets with a meal, three times a day, for a total of 6 tablets per day. Products were manufactured by Standard Process (Palmyra, WI, USA). Participants were required to record their product intake in a study diary. Compliance was measured by counting the returned study tablets at each visit. The percent compliance was calculated by determining the number of dosage units taken, divided by the number of dosage units expected to have been taken, multiplied by 100. In the event of a discrepancy between the information in a participant’s diary and the amount of study product returned, the compliance was calculated based on the product returned unless a reasonable explanation for the discrepancy was provided. Participants with compliance less than 80% or greater than 120% were counselled to improve compliance.

### 2.4. Randomization and Blinding

A block randomization schedule was prepared by one un-blinded staff member not involved in any study assessment. Within each block of 4 consecutively enrolled participants, 2 received A-F Betafood^®^ and 2 received a placebo in a random order generated using randomization.com. Upon enrollment, every participant was assigned a unique randomization number based on the randomization schedule. Treatment allocation was implemented using 6-digit randomization codes, with the list generated by an un-blinded KGK staff member who was not involved in conducting the study. In the case of a reported serious adverse event that required the randomization code to be broken for that individual, the associated treatment for each participant was sealed in an opaque envelope and labelled with the participant randomization number.

### 2.5. Outcomes

The primary outcomes assessed gallbladder and liver function at baseline and 12 weeks post-supplementation. Gallbladder function was determined by ultrasound measurements of gallbladder volume, GBEF, GBER, and wall thickness. Liver function was assessed using standard laboratory tests for AST, ALT, and GGT. Inflammation was determined by measuring hsCRP. The secondary outcomes assessed total cholesterol, HDL-C, LDL-C, triglycerides, oxLDL, MDA, TNF-α, and adiponectin concentrations. All participants were subject to safety assessments including vital signs, BMI, complete blood counts (CBC), electrolytes, creatinine, and total bilirubin. Participants were asked to report the incidence of any adverse events (AE) as defined by the Medical Dictionary for Regulatory Activities version 17.0 and reported concomitant medications in a treatment diary.

### 2.6. Laboratory Measurements

Fasting gallbladder volume, GBEF, GBER, and wall thickness were measured using ultrasound at My Health Center, London, ON, Canada and SimonMed Imaging—Downtown Kaley, Orlando, FL, USA. To measure post-prandial GBEF and GBER, participants consumed a high fat liquid meal (BOOST Plus^®^) and 1 tablespoon of fluid whipping cream (Nielson) to induce gallbladder emptying, and an ultrasound was taken at 5-min intervals for 30 min. 

Whole blood was collected into EDTA tubes for CBC analysis. Serum was obtained for the analysis of electrolytes, glucose, blood urea nitrogen (BUN), creatinine, eGFR, AST, ALT, GGT, bilirubin, hsCRP lipid profile oxidized LDL (oxLDL), malonyldialdehyde (MDA), TNF-α, and adiponectin. Aliquots of serum were transferred into cryovials and stored at −40 °C. Additionally, baseline serum samples for all women of child-bearing potential were analyzed for hCG. The total blood volume collected for the laboratory assessments listed above will be approximately 65 mL, over the period from screening to end of study. At any study visit, blood loss per subject was not expected to exceed 30 mL.

Laboratory tests for AST, ALT, GGT, hsCRP, total cholesterol, HDL-C, LDL-C, and triglycerides and safety assessments were analyzed at Life Labs, London, ON, Canada and LabCorp, Irvine, CA, USA; Orlando, FL, USA using standard procedures. Analysis of AST, ALT, GGT, hsCRP, and lipids were analyzed using Roche cobas^®^ c 701 analyzer (Mississauga, ON, Canada). Analysis of oxLDL, MDA, and adiponectin were measured at Eve Technologies (Calgary, AB, Canada) using ELISA methodology. TNF-α was measured at Eve Technologies (Calgary, AB, Canada) using Luminex methodology.

### 2.7. Statistical Analysis

Power calculations for the study were performed based on the GBEF as the primary endpoint with a predicted effect size difference of 7% between groups. An 80% power and α = 0.05 with an attrition rate of 15% led to a target sample size of 50 participants, which consisted of overweight but otherwise healthy participants. Student *t*-tests were performed to assess changes in GBEF after 12 weeks of supplementation. Paired Student *t*-test was used for comparing changes in endpoints from baseline to week 12 within groups. Unpaired Student *t*-test was used for comparing changes in endpoints at week 12 between placebo and A-F Betafood^®^. Numerical endpoints were tested for significance between groups by Analysis of Covariance (ANCOVA). Categorical endpoints were tested for significance between groups by Fisher’s exact test. Changes in endpoints were significant at α = 0.05 (*p* ≤ 0.05). All statistical evaluations were performed using R Statistical Software Package Version 3.0.3. (R Core Team, 2014) for Microsoft Windows.

## 3. Results

### 3.1. Subjects 

**Figure 2:**
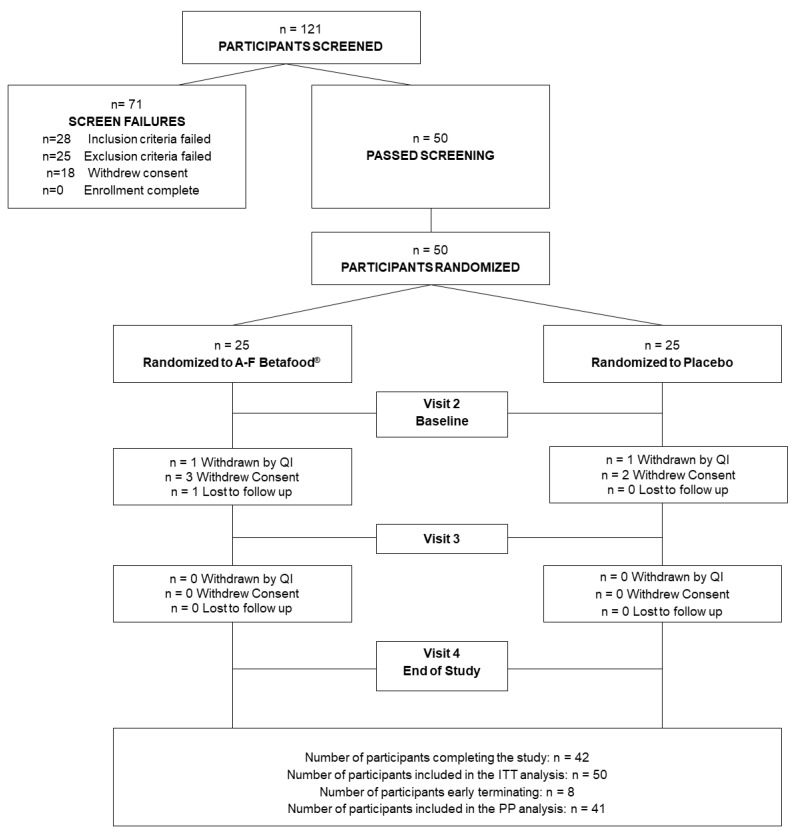
Participant disposition. Of the 121 participants screened, 50 eligible participants were enrolled and were randomized to receive either A-F Betafood^®^ or placebo at 25 participants per group. Eight participants, all early terminations, were not included in the PP population. “Withdrawn by QI” refers to a participant who is removed from the study by the Qualified Investigator for breach of the study protocol. “Withdrew consent” refers to a participant who withdraws themselves (or discontinues) from the study for personal reasons. ITT, intention to treat; PP, per protocol.

A total of 50 eligible and consenting participants (mean age 54.25 ± 8.01 years; mean BMI 27.59 ± 2.05 kg/m^2^) were randomized equally in a 1:1 ratio to receive either A-F Betafood^®^ or placebo. All participants were deemed healthy by the QI as per the assessment of vital signs, hematology, clinical chemistry, and physical examination. The demographics between the A-F Betafood^®^ and placebo groups were similar (Table 1). The Per Protocol (PP) population comprised of 42 participants that consumed at least 80% of either product dose, who did not have any major protocol violations, and completed all study visits and procedures required for the analysis of the primary outcome. The study compliance for participants in the A-F Betafood^®^ and placebo groups were 97.1% and 96.1%, respectively. A total of five participants in the A-F Betafood^®^ group and three participants in the placebo group dropped out of the study (Figure 2).

### 3.2. Primary Outcomes

#### 3.2.1. Gallbladder Volume

**Figure 3:**
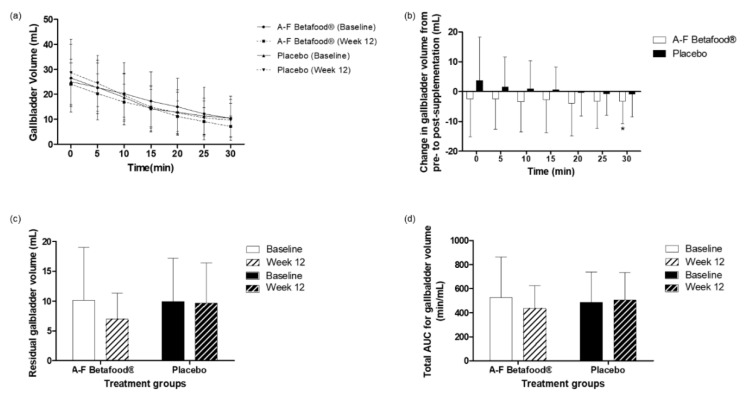
Ultrasound gallbladder volume assessments in the per protocol population at baseline and week 12 post-supplementation with A-F Betafood^®^ or placebo (n = 42). (**a**) Mean gallbladder volume and (**b**) mean change in gallbladder volume measured at five-min intervals, (**c**) mean residual gallbladder volume and (**d**) total AUC for gallbladder volumes. Mean ± SD, where * *p* < 0.05. AUC, area under the curve.

There were no significant differences between the A-F Betafood^®^ and placebo groups in the mean change in gallbladder volume (Figure 3a) or mean gallbladder residual volumes (Figure 3c). The total gallbladder volume area under the curve (AUC) accounts for both fasting and post-prandial gallbladder volumes and is representative of gallbladder emptying over the 30-min testing period. The changes in the gallbladder volume AUC from baseline to week 12 were not significantly different between the A-F Betafood^®^ and placebo groups (Figure 3d).

A number of changes were observed within the A-F Betafood^®^ group: a reduction in the mean gallbladder volume at all time points with the difference at 30 min reaching statistical significance (*p* = 0.044) (Figure 3b); a 31% reduction in the mean gallbladder residual volume (*p* = 0.058) (Figure 3c); and a tendency for a decrease in gallbladder AUC (Figure 3d).

#### 3.2.2. GBEF

**Figure 4:**
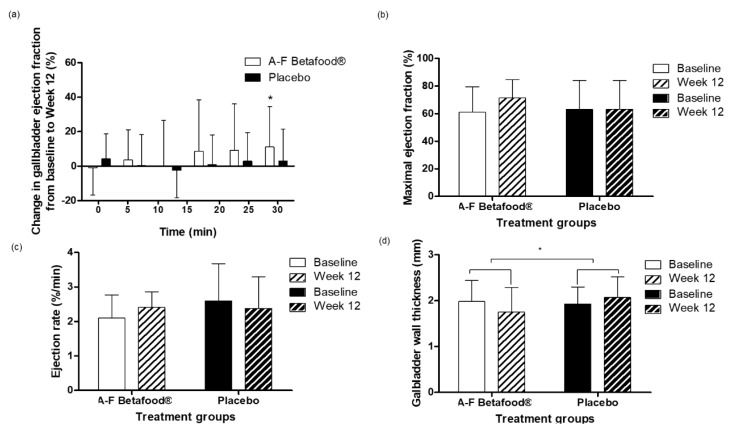
Gallbladder function in the per protocol population at baseline and week 12 post-supplementation with A-F Betafood^®^ or placebo (n = 42). (**a**) Mean change in ejection fraction, (**b**) mean maximal ejection fraction, (**c**) mean ejection rate, and (**d**) mean gallbladder wall thickness. Mean ± SD, where * *p* < 0.05. mm, millimeter.

There was no significant difference between the A-F Betafood^®^ and placebo groups in change in GBEF (Figure 4a), while the change in maximal GBEF tended to be higher (*p* = 0.072) in the A-F Betafood^®^ group as compared to the placebo group.

Within the A-F Betafood^®^ group, 12-week supplementation resulted in a significant 19% increase compared to baseline (*p* = 0.047) (Figure 4a) and a 17.1% increase in maximal GBEF (*p* = 0.055) (Figure 4b).

#### 3.2.3. GBER

There were no significant between- or within-group differences in change in GBER from baseline to week 12 (Figure 4c).

#### 3.2.4. Gallbladder Wall Thickness

There was no significant difference in gallbladder wall thickness between the A-F Betafood^®^ and placebo groups. However, the change in the gallbladder wall thickness from baseline to week 12 was significantly different between the two groups (*p* = 0.049) due to a 9% decrease from baseline in the A-F Betafood^®^ group and a 7% increase in the placebo group (Figure 4d).

#### 3.2.5. AST, ALT, GGT, hs-CRP

**Figure 5:**
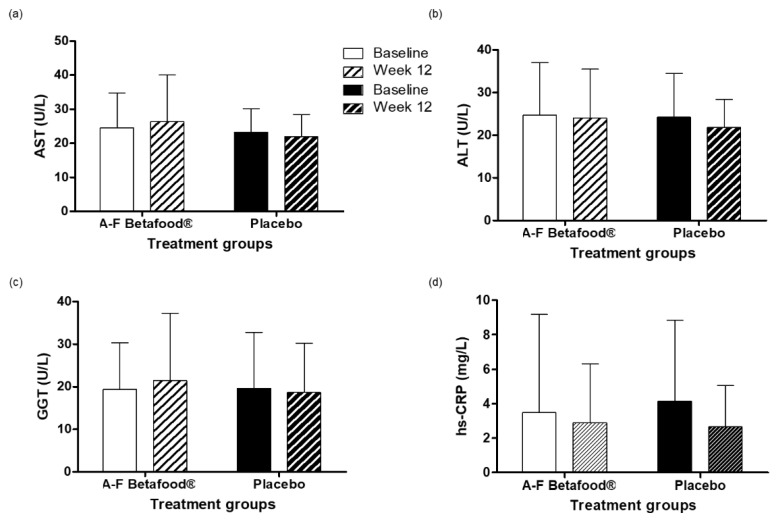
Liver function and inflammatory markers in the per protocol population at baseline and week 12 post-supplementation with A-F Betafood^®^ or placebo (*n* = 42). (**a**) AST, (**b**) ALT, (**c**) GGT and (**d**) hs-CRP. Mean ± SD. AST, aspartate aminotransferase; ALT, alanine aminotransferase; GGT, gamma-glutamyltransferase; hs-CRP, high-sensitivity C-reactive protein.

The change in liver function markers (AST, ALT, and GGT) and hs-CRP concentrations from baseline to week 12 were not significantly different within or between the A-F Betafood^®^ and placebo groups (Figure 5a–d).

### 3.3. Secondary Outcomes

#### oxLDL, MDA, TNF-α, and Adiponectin

**Figure 6:**
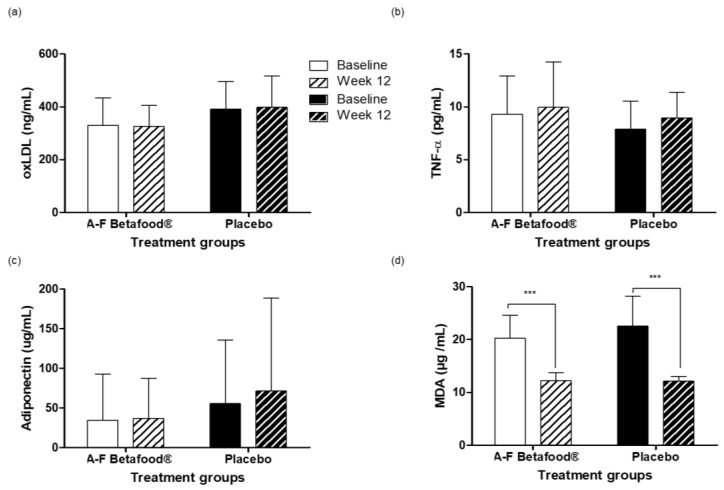
Oxidative stress, inflammatory, and gallbladder health markers in the per protocol population at baseline and week 12 post-supplementation with A-F Betafood^®^ or placebo (*n* = 42). (**a**) oxLDL, (**b**) TNF-α, (**c**) adiponectin and (**d**) MDA. Mean ± SD, where *** *p* < 0.001. TNF-α, tumor-necrosis factor—α; MDA, Malonyldialdehyde; oxLDL, oxidized low-density lipoprotein. 3.3.2. Total Cholesterol, LDL-C, HDL-C, Triglycerides

There were no significant between- or within-group differences in the oxLDL, TNF-α or adiponectin concentrations from baseline to week 12 (Figure 6a–c). However, within-groups, both A-F Betafood^®^ and the placebo showed significant decreases (*p* < 0.001) in MDA concentrations at week 12 compared to the baseline concentrations (Figure 6d).

**Figure 7:**
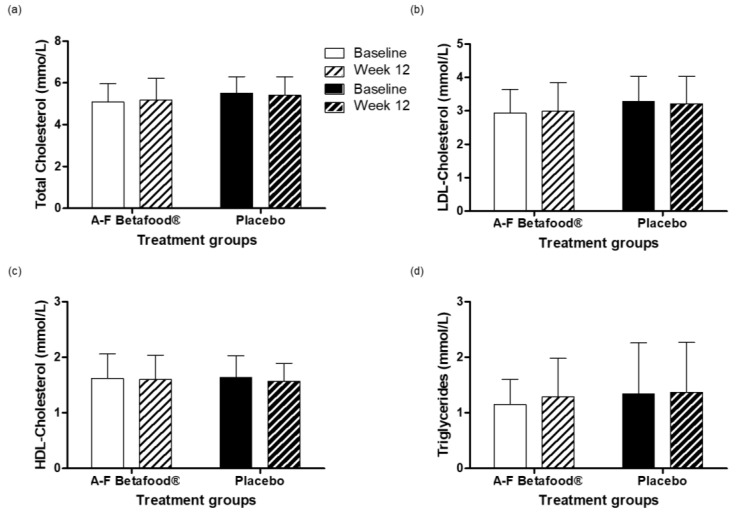
Lipid profile in per protocol population at baseline and week 12 post-supplementation with A-F Betafood^®^ or placebo (*n* = 42). (**a**) Total Cholesterol, (**b**) LDL-Cholesterol (**c**) HDL-Cholesterol and (**d**) triglycerides levels. Mean ± SD. LDL, low-density lipoprotein; HDL, high-density lipoprotein.

There were no significant differences within or between the A-F Betafood^®^ and placebo groups in the total cholesterol, LDL-C, HDL-C, or triglycerides concentrations from baseline to week 12 (Figure 7).

### 3.4. Adverse Events

Fifty AE were reported by 24 participants in this study. Of these, 13 AE were reported by eight participants who received A-F Betafood^®^ and 37 AEs were reported by 16 participants who received the placebo. The 13 AE reported by participants consuming A-F Betafood^®^ were: nausea, indigestion, increased bowel peristalsis, pale feces, heartburn, hyperlipidemia, low heart rate, flu, stomach flu, heat stroke, runny nose, chest cold and Cullen’s sign. The AE categorized as probably related was heartburn; AEs categorized as possibly related were nausea, pale feces, indigestion; the rest of the AEs were categorized as not related or unlikely related. The 37 AE reported by participants consuming the placebo were: cholangitis, heart burn, flu, GI upset, common cold, stomach pain, flatulence, gallbladder pain, headache, frequent urination, itchy skin, bronchitis, constipation, ageusia, diarrhea, muscle strain, cramps, chills, hives, hair loss, varicose veins, and hyperlipidemia. The AE categorized as possibly related was gallbladder pain; the rest of the AE were categorized as not related or unlikely related. All AE were resolved by end of study, and no participants required medical treatment or hospitalization. There were no reported serious AE.

## 4. Discussion

This randomized, double-blind, placebo-controlled parallel study supports the use of A-F Betafood^®^ in promoting gallbladder function. Gallbladder disease severely impairs patient health and cholecystectomy is currently the most common and definitive medical management approach in many cases [4]. Given the significant economic burden associated with gallbladder function, the discovery of a preventative approach is in high demand [15]. Gallbladder wall thickness influences motility of the gallbladder, with a reduction in wall thickness leading to improved functionality [16]. Participants enrolled in this study had a gallbladder wall thickness of less than 3 mm, as wall thickness greater than 3 mm is associated with impaired gallbladder function [16]. In this study, consumption of A-F Betafood^®^ for 12 weeks resulted in a significant reduction in gallbladder wall thickness compared to placebo. Supplementation with A-F Betafood^®^ resulted in a 9% decrease in gallbladder wall thickness, whereas the placebo group showed a 7% increase. This data suggests that A-F Betafood^®^ supplementation may improve gallbladder motility through reduced wall thickness. 

Gallbladder volumes in healthy individuals and individuals with gallbladder disease are vastly different, therefore, gallbladder volume is a measure of gallbladder function [17]. The fasting gallbladder volume in healthy adults is much lower than in individuals with gallstone disease [17,18]. Although there were no significant changes between groups, observational data showed that supplementation with A-F Betafood^®^ for 12 weeks resulted in a 9% decrease in mean fasting volume, whereas the placebo resulted in a 15% increase. Additionally, a 12-week supplementation with A-F Betafood^®^ resulted in a significant reduction in mean gallbladder volume 30 min following consumption of the high-fat liquid meal. Gallbladder AUC accounts for both the fasting and post-prandial gallbladder volume following the consumption of a high-fat liquid meal. A-F Betafood^®^ resulted in a non-significant 18% decrease in the gallbladder volume AUC. These observations suggest potential improvements in gallbladder functioning by supplementing with A-F Betafood^®^.

High fasting gallbladder volume can be an indicator of impaired gallbladder motility [18]. Reduced gallbladder motility results in impaired gallbladder emptying and leads to sludge formation, crystal nucleation as well as stone formation [3]. GBEF and GBER are some of the most commonly accepted measures of evaluating gallbladder motility with an increase in either measurement representing improved gallbladder motility [19]. Although there were no significant changes between groups, observational data showed that supplementation with A-F Betafood^®^ led to a significant 19% within-group increase in the GBEF at 30 min, compared to the placebo group that showed a 5% increase. Further observational data showed that the A-F Betafood^®^ group exhibited a 14% increase in GBER, whereas the placebo resulted in an 8% decrease. Taken together these observational data suggest that A-F Betafood^®^ supplementation improves gallbladder motility. This is supported by the results above that show improvements in gallbladder wall thickness after A-F Betafood^®^ supplementation.

Gallbladder function can directly and indirectly determine liver function and manifest in the form of increased levels of transaminases [20], therefore, liver function tests were performed. Supplementation with A-F Betafood^®^ did not yield any significant changes in any of the liver parameters tested and remained within clinically normal ranges for both groups. Individuals suffering from gallstone disease also present markedly different lipid levels than healthy participants [21]. No significant changes in total plasma cholesterol, LDL-C, HDL-C, and triglycerides were found during the study period. Furthermore, supplementation with A-F Betafood^®^ showed no significant differences in the oxLDL, adiponectin or TNF-α concentrations, indicating that consumption does not result in lipoprotein abnormalities or inflammation. Moreover, a significant reduction in the concentrations of MDA, an end-product of lipid peroxidation, in both the A-F Betafood^®^ and placebo groups is suggestive of decreased oxidative stress and in turn a decrease in inflammation. One of the major ingredients in A-F Betafood^®^ is beet leaf, rich in nitrite and nitrate. Studies have shown that nitrate and nitrite preserve vascular function [22]. In addition, dietary nitrate has been shown to improve blood circulation by the preservation of vascular endothelial functions [23].

Several medications such as erythromycin, ursodeoxycholic acid, and non-steroidal anti-inflammatory drugs (NSAIDs) are pre-surgical therapies currently used to improve gallbladder motility [24]. In healthy adults, erythromycin administered orally or intravenously improves gallbladder motility by significantly decreasing both fasting and post-prandial gallbladder volumes [24]. Catnach et al. demonstrated that a single bolus dose of 500 mg erythromycin administered orally resulted in a significant 30% and 51% reduction in fasting and residual gallbladder volumes respectively [25]. NSAIDs also have been reported to confer improvements in gallbladder motility, however, the effect appears to be restricted to patients with gallstones [24]. Overall, these medications are recommended to be used as therapeutic agents after the diagnosis of gallbladder disease, and long-term consumption of these drugs is associated with various side effects. 

This study had shown an improvement in the gall bladder function as observed by reduced fasting gallbladder and residual volumes in participants with gall bladder conditions. Although this study was adequately powered to detect the anticipated effect size, the population studied was relatively homogenous, consisting predominately of Caucasian (88%–96%) women (76%). Further, the participant recruitment process was also slow as this was a unique population to enroll. The current management strategy for gallbladder issues is the removal of the gallbladder, thus it was challenging to find individuals who were eligible who had not sought or were currently seeking treatment. A larger and more diverse population will also allow examination of the effect of sex or ethnicity on efficacy of the product [26]. Additionally, there was a lack of information on the family history of gallbladder insufficiency in this at-risk population, which has been shown to increase the risk of disease development by nearly five-fold [26]. Thus, these limitations have to be considered when generalizing the findings of this study.

In conclusion, data from this trial suggest improvements in markers of gallbladder function following supplementation for 12 weeks with A-F Betafood^®^, a whole food-based health product. In the absence of any adverse effects, A-F Betafood^®^ supports a healthy gallbladder function in the individuals at risk of gall bladder insufficiency. 

## Figures and Tables

**Table 1:** Demographics for all participants enrolled (n = 50).

	A-F Betafood^®^ (*n* = 25)	Placebo (*n* = 25)	Between Group *p*-Value ^1^
Age (years)	54.28 ± 8.21	54.23 ± 7.97	0.98
Gender (*n* (%))			1.00
Female	19 (76.00%)	19 (76.00%)	
Male	6 (24.00%)	6 (24.00%)	
Systolic BP (mmHg)	114.16 ± 12.26	117.40 ± 11.28	0.34
Diastolic BP (mmHg)	72.88 ± 9.70	73.08 ± 8.76	0.94
Heart Rate (bpm)	67.25 ± 8.13	8.40 ± 7.98	0.62
BMI (kg/m^2^)	27.24 ± 2.05	27.95 ± 1.88	0.20
Race (n (%))			0.15
Black or African American	1 (4.00%)	0 (0.00%)	
Eastern European White	6 (24.00%)	1 (4.00%)	
North American Indian	1 (4.00%)	0 (0.00%)	
South American	1 (4.00%)	1 (4.00%)	
Western European White	16 (64.00%)	23 (92.00%)	

N/n, number; %, percentage; SD, standard deviation. ^1^ For continuous parameters, between group *p*-values were generated from ANOVA models with Group as a fixed effect. For categorical parameters, between group *p*-values generated by Chi-square or Fisher’s Exact (2-tail) tests as appropriate.
